# Exploring the Italian equine gene pool *via* high-throughput genotyping

**DOI:** 10.3389/fgene.2023.1099896

**Published:** 2023-01-23

**Authors:** Stefano Capomaccio, Michela Ablondi, Daniele Colombi, Cristina Sartori, Andrea Giontella, Katia Cappelli, Enrico Mancin, Vittoria Asti, Roberto Mantovani, Alberto Sabbioni, Maurizio Silvestrelli

**Affiliations:** ^1^ Department of Veterinary Medicine, University of Perugia, Perugia, Italy; ^2^ Sport Horse Research Centre (CRCS), University of Perugia, Perugia, Italy; ^3^ Department of Veterinary Science, University of Parma, Parma, Italy; ^4^ Department of Agricultural, Food, and Environmental Sciences, University of Perugia, Perugia, Italy; ^5^ Department of Agronomy, Food, Natural Resources, Animals, and Environment, University of Padua, Padua, Italy

**Keywords:** biodiversity, local breeds, genomic data, SNP, horse

## Abstract

**Introduction:** The Italian peninsula is in the center of the Mediterranean area, and historically it has been a hub for numerous human populations, cultures, and also animal species that enriched the hosted biodiversity. Horses are no exception to this phenomenon, with the peculiarity that the gene pool has been impacted by warfare and subsequent “colonization”. In this study, using a comprehensive dataset for almost the entire Italian equine population, in addition to the most influential cosmopolitan breeds, we describe the current status of the modern Italian gene pool.

**Materials and Methods:** The Italian dataset comprised 1,308 individuals and 22 breeds genotyped at a 70 k density that was merged with publicly available data to facilitate comparison with the global equine diversity. After quality control and supervised subsampling to ensure consistency among breeds, the merged dataset with the global equine diversity contained data for 1,333 individuals from 54 populations. Multidimensional scaling, admixture, gene flow, and effective population size were analyzed.

**Results and Discussion:** The results show that some of the native Italian breeds preserve distinct gene pools, potentially because of adaptation to the different geographical contexts of the peninsula. Nevertheless, the comparison with international breeds highlights the presence of strong gene flow from renowned breeds into several Italian breeds, probably due to historical introgression. Coldblood breeds with stronger genetic identity were indeed well differentiated from warmblood breeds, which are highly admixed. Other breeds showed further peculiarities due to their breeding history. Finally, we observed some breeds that exist more on cultural, traditional, and geographical point of view than due to actual genetic distinctiveness.

## Introduction

Despite being domesticated thousands of years after dogs, cattle, pigs, and other mammals (Frantz et al., 2015; Park et al., 2015) (i.e., approximately 5.500 years ago), the horse (*Equus ferus caballus*) rapidly became the most important animal in the development of human civilization. At first, horses were used for meat and milk production, but after the invention of horse collars and horseshoes, they were employed as raw power in agriculture, a role that was maintained until the industrial revolution. Horse’s domestication also guaranteed a “technological” advantage in ancient times due to their use in transportation, allowing the spread of populations, language, culture, religion, goods, trades, and also diseases (Librado et al., 2016). Horses have been, for thousands of years, man’s loyal companions in warfare and conquest.

For centuries, humans applied a strong selective pressure on this species, differentiating breeds and types for specific characteristics, such as performance, aesthetics, coat color, or adaptation to the local environment, until the complete extinction of wild horses (McCue et al., 2012; Librado et al., 2016; Librado et al., 2021). In the past 400 years, artificial selection of horses has reached its peak and modern breeds have emerged, whereas geographic distribution has played a predominant role in animal evolution and breed differentiation. This long and incessant selection process has led to a dramatic loss of genetic diversity in the past 200 years, while it had remained almost unaltered in the past thousands of years (Fu and Li, 2022). Y chromosome diversity loss began much earlier because of the preference to use a few selected stallions in bloodline development (Wallner et al., 2013), with more substantial and ancient loss of genetic diversity. High percentages of inbreeding coefficients and low heterozygosity levels were associated with this process.

The Italian peninsula is a heterogeneous territory with many ecological niches that encompass various climates, cultures, and their combinations. This diversity has been reflected also in the FAO (https://www.fao.org/dad-is/data/en/) report where 30 different breeds are reported in the peninsula. Of these, more than 20 are considered autochthonous; however, their origins and relationships remain unclear. The horses bred in the Italian territory can be divided into two main types: coldblood (height at the withers 148–165 cm; mainly used for heavy work in the northern regions), and warmblood (height at the withers 148–170 cm; preferred in light work and equestrian sports, mainly bred in the Central and Southern regions of the country). Insular and harsh regions, where extensive breeding is preferred, have experienced selection towards smaller size (height at withers 115–147 cm) (Cardinali et al., 2016). Until World War II, Italian horses were bred mostly for military and agricultural purposes. After the war, a dramatic drop in population and a bottleneck in the equine genetic pool occurred, followed by an industrialization process that made the traditional role of horses obsolete. Thus, the main interest in the equine industry remained in sports, culture, and riding. Nevertheless, most Italian breeds are endangered due to decades of decreased interest in equine breeding. Genetic diversity loss is also a major problem, exacerbated by the small population size, which makes conservation projects both challenging and expensive to build and sustain but is eased by the use of genomic technology (Eusebi et al., 2020). During the last few decades, some efforts have been made to disentangle the relationship between the Italian equine population and low-density (Bruzzone et al., 2003; Zuccaro et al., 2008; Felicetti et al., 2010; Bigi and Perrotta, 2012) or genomic data (Criscione et al., 2015; Ablondi et al., 2020a; Mancin et al., 2020; Criscione et al., 2022) were produced, but a clear picture of the entire genetic variability is not yet available. This study aimed to understand the origins and current genetic status of the Italian equine population.

## Materials and methods

### Samples

Samples from Italian breeds collected for this study were part of the PSRN project. Authorized veterinarians with Ministry approval (PSRN 2014-2020—10.2, ID: J59H18000030005; Approval D.M. 6695, 21/02/2018) collected hair bulbs for DNA extraction performed at AGROTIS S.R.L.—LABORATORIO GENETICA E SERVIZI (Cremona, Italy). Depending on the breed head count, different information was considered: pedigree data obtained from the National Horse Breeders Associations or family-based information for minor breeds. Genotyping was performed in the same laboratory by using the GGP Equine 70 k ^®^ array. Here, we represented the Italian equine diversity by collecting 1,308 individuals from 22 Italian horse populations, genotyped at 65 157 SNPs. This dataset was then merged with publicly available data from Petersen and colleagues (Petersen et al., 2013) with 32 of the most relevant international breeds and 54 602 genotyped SNPs.

### Dataset creation and quality control

Two main working datasets were defined. The first, containing only Italian breeds, was used to investigate the genetic relationships within the pure Italian gene pool. The second dataset grouped both Italian and international breeds (here named the International dataset) to evaluate the inward and outward gene flow, even from the Italian peninsula. Dataset management was performed using PLINK v1.9 (Purcell et al., 2007). Quality control (QC) was carried out using the BITE 1.2.0008 package in R (Milanesi et al., 2017), removing all unfitting individuals and markers according to the following thresholds: ID call rate of 0.90, SNP call rate of 0.95, individual call rate 0.95, HW eq. FDR = 0, IBS threshold 0.95, and minor allele frequency (MAF) of 0.05. Linkage Disequilibrium (LD) pruning was not performed since no differences in terms of MDS and Admixture results were found when performing a hard LD pruning (indep-pairwise 50 5 0.2). For the Italian dataset, due to the large differences in breeds’ sample sizes, the maximum number of IDs for each breed was limited to 50, with a supervised subsampling using the procedure of the function *representative.sample()* in BITE (Milanesi et al., 2017). To define if the subsample was representative of the total group, a similarity threshold of 0.65 was set and the maximum number of trials—intended as an attempt to find a suitable subset—was 5 000.

### Multidimensional Scaling (MDS), admixture, gene flow, and effective population size (N_e_)

In the case of Multidimensional Scaling (MDS) and admixture, both Italian and International datasets were analyzed. MDS was realized in BITE through the function *bite.biostat()* and the first six dimensions were evaluated. Population structure analyses were conducted using the ADMIXTURE software (Alexander et al., 2009), and the applied *K* value (number of subpopulations) ranged from 2 to 30 for the Italian dataset and from 2 to 60 for the International dataset. ADMIXTURE also allows a cross-validation procedure using the flag*--cv*, which estimates the accuracy of ancestry percentages, and the lowest estimated value of cross-validation (CV) error is associated with the most fitting value of ancestral genomes.

Potential migration events were investigated using TreeMix 1.13 software (Pickrell and Pritchard, 2012) hypothesizing a maximum of 20 migrations, and using a window of 250 consecutive SNP blocks. The analysis was repeated 3 times in order to calculate the optimal *m* value (migration event number). The most fitting *m* value was evaluated using the OptM package (Fitak et al., 2019) implemented in *R* with the *optM()* function, resulting in *m* = 8. Donkeys genotyped using the same chip were available and added for this specific analysis. A total of 35 random individuals from major Italian breeds were used for migration analysis and rooting of the tree-based graph output.

Finally, nodes likelihood was assess through 100 bootstrap repetitions at the most fitting *m* value, and a consensus tree was produced using PHYLIP software (Felsenstein, 1993). Finally, trends in effective population size (Ne) were evaluated using SNeP v 1.1 software (Barbato et al., 2015). Only breeds with a minimum consistency of 40 genotyped animals (before subsampling) were analyzed to allow correct Ne estimation (personal communication with the SNeP author). All output results are plotted in R v. 4.0.5.

## Results

### Dataset creation and quality control

After QC, 52 445 of the initial 65 157 SNPs were considered for the Italian dataset and 43 854 of the initial 54 602 for the international dataset. We pooled the two resulting datasets in a combined dataset with 34,544 SNPs and counting 1,333 individuals of which 608 were Italian and used for the demographic analysis (Supplementary Table S1).

### Multidimensional Scaling

**FIGURE 1:**
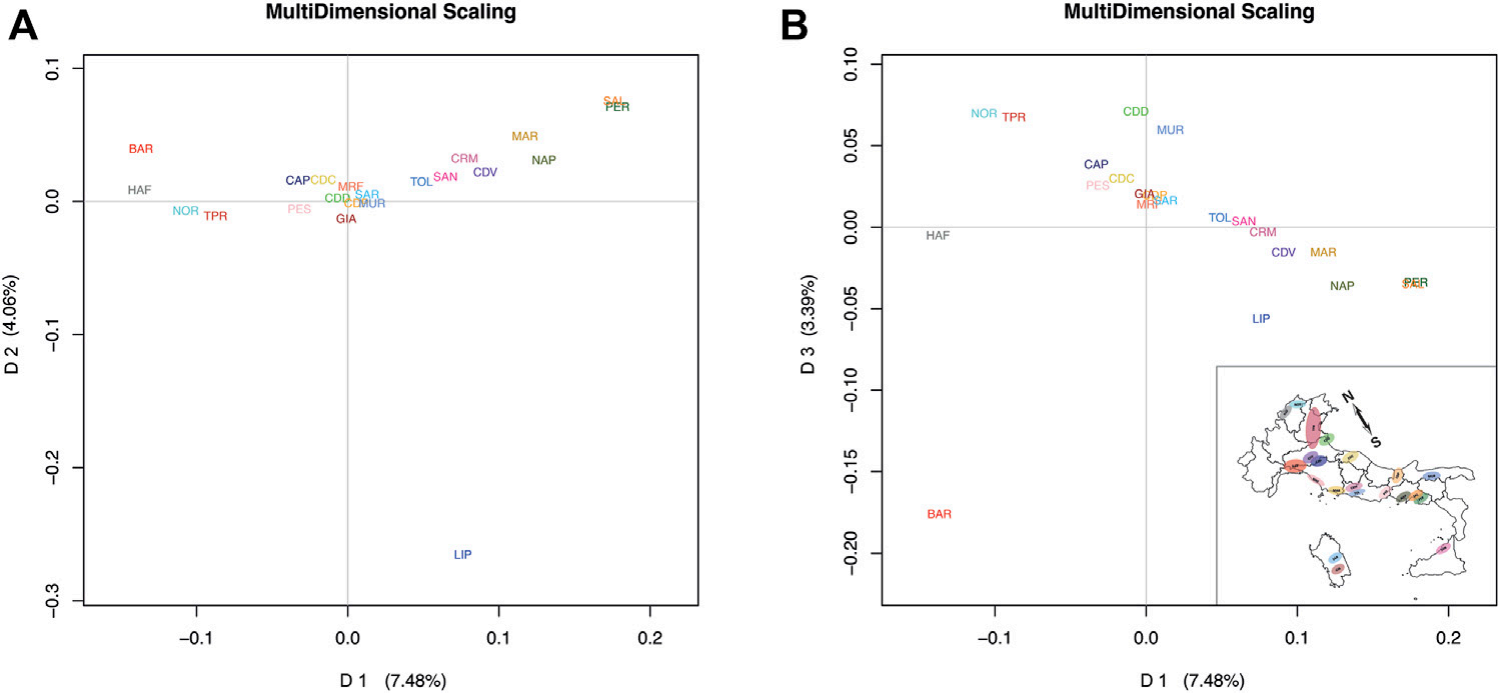
MDS plots of average coordinates for each breed for the Italian dataset. Full list of used acronyms is available in Supplementary Table S1. **(A)** D1 *vs*. D2 plot based on Italian dataset analysis. **(B)** D1 *vs*. D3 plot mirroring the geographic Italian breeds distribution in the country. The Italian map graph is rotated for higher clarity.

**FIGURE 2:**
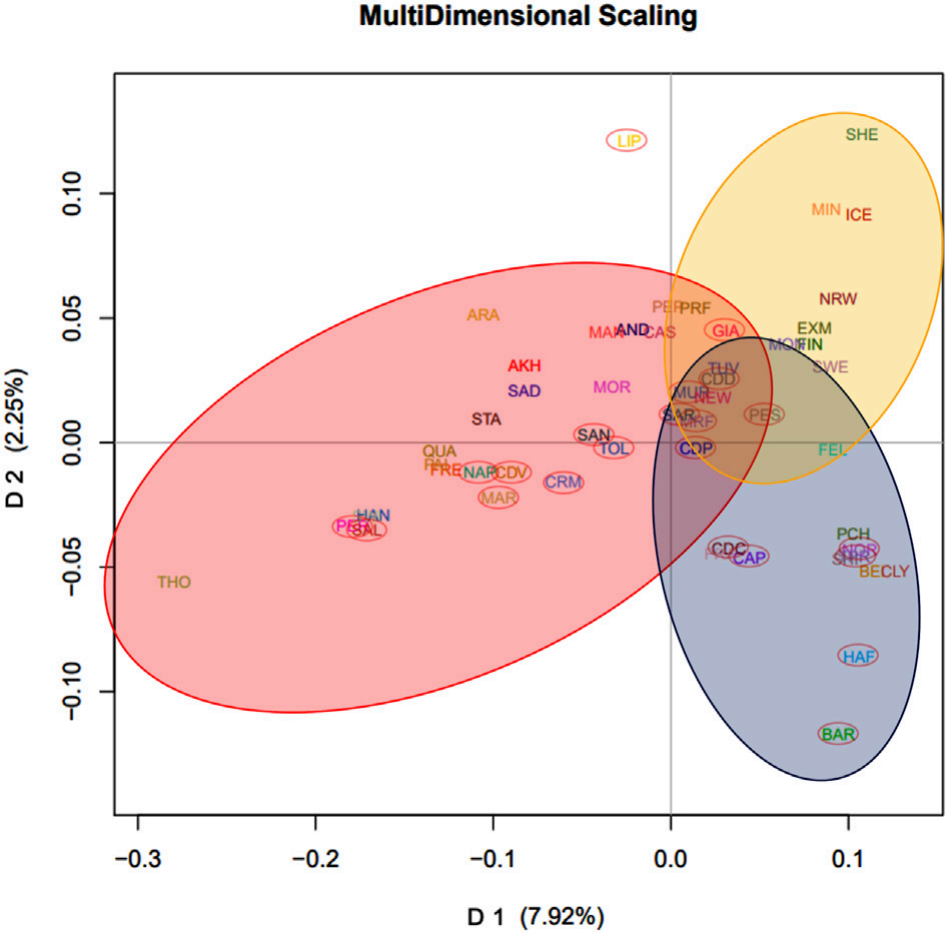
MDS plots of average coordinates of dimension 1 (D1) and 2 (D2) for the International dataset. D1 reflects the warmblood quantity while D2 discriminates ponies. Three clusters were highlighted: warmblood horses, circled in red, coldblood horses, circled in blue, and ponies, circled in yellow. Italian breeds were also highlighted for clarity with a red ellipse.

In the Italian dataset, the first dimension (D1) explained 7.48% of the total variance, whereas D2 explained 4.06%. Plotting D1 *vs*. D2 (Figure 1A), a breed division along the first dimension following a North-to-South geographical distribution can be distinguished according to the proportion of warmblood ancestry, while the second dimension discriminates the Lipizzaner (LIP) breed from the others, showing that LIP is a unique breed. The third dimension, which explained 3.39% of the variance, discriminates breeds from East to West by geographical distribution. By plotting D1 *vs*. D3, a genetic distribution that loosely recalls actual geographic distribution of the breeds can be observed (Figure 1B). We noted that our MDS of the Italian dataset separated breeds according to spatial distribution and warmblood ancestry: Bardigiano (BAR) horse is found in the upper-left portion of the graph, being its area between Liguria, Emilia-Romagna and Tuscany, Noriker (NOR), and Haflinger (HAF) horse, breeds originating at the border with Austria, are in the upper portion, the Italian Heavy Draught Horse, which Italian name is Cavallo Agricolo Italiano da Tiro Pesante Rapido (TPR) in the upper-right, Veneto the origin area, and Cavallo del Delta (CDD) and Murgese (MUR) on the right of the graph, being bred in the eastern parts of Italy. Other breeds follow the Italian continental axis but show less accuracy in the geographic-based distribution, probably because of their higher levels of admixture. In contrast, for the International dataset (Italian plus international breeds), D1 explained 7.92% of the genetic variance and reflected the warmblood quantity in each breed. D2, which explained 2.25% of the genetic variance, distinguished ponies from other breeds. By plotting D1 *vs*. D2 (Figure 2), the division between warmblood, coldblood, and pony clusters is clear. Within this dataset, Italian breeds, with a few exceptions, appear even more admixed; the Italian breeds that show more identity are the LIP, which is completely isolated from the three clusters, and the coldblood BAR and HAF horses. Most Italian breeds fall in the warmblood cluster, and many of them, such as CDD, MUR, Sarcidano (SAR), Monterufolino (MRF), Pony Esperia (PES), and Cavallo Pentro (CDP), are at the intersection of the three clusters. Further separation of MUR and CDD can be appraised as D4 and D5 in the Italian dataset analysis (Supplementary Figure S1).

### Structure analysis

**FIGURE 3:**
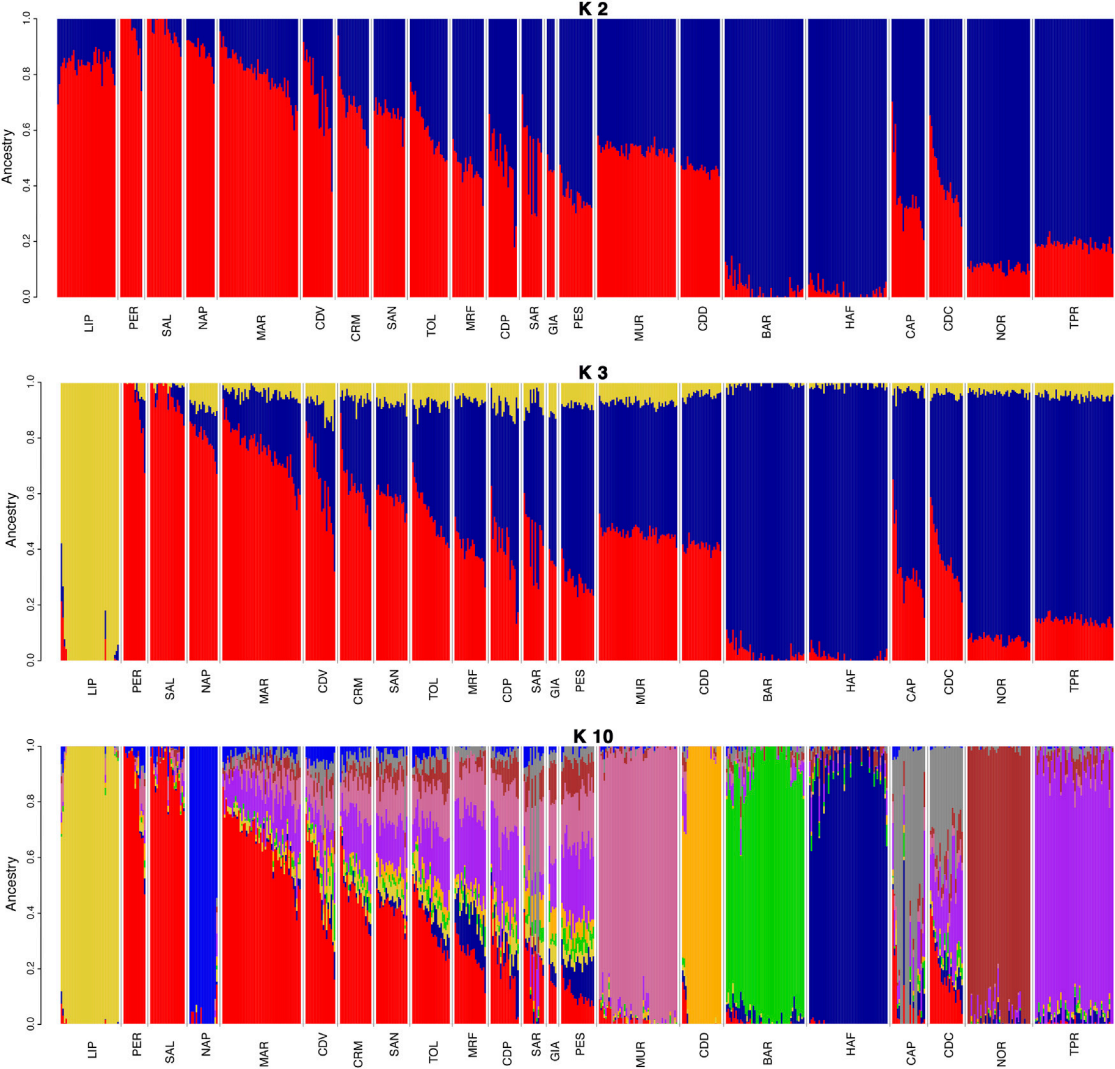
Admixture plot with K values 2, 3, and 10 for the Italian dataset. The clustering of Bardigiano (BAR) occurs at K = 4, Cavallo del Delta (CDD) at K = 5, Cavallo Agricolo Italiano da Tiro Pesante Rapido (TPR) at K = 6, Murgese (MUR) at K = 7, Noriker (NOR) at K = 8, Monterufolino (MRF) at K = 9, and Napoletano (NAP) at K = 10. Full list of used acronyms is available in Supplementary Table S1.

The population structure analysis of the Italian dataset (Figure 3) was performed for K values from 2 to 30 (Supplementary Figure S2), with K = 19 being the lowest estimated cross-validation (CV) error (Supplementary Figure S3). At K = 2, a clear separation of warmblood (in red) and coldblood (in blue) horses was observed, with a generally higher level of warmblood ancestry in southern and central Italian breeds. By increasing the *K* value to 3, we observed an early separation (in yellow) of LIP, confirming the MDS results. *K* values from 4 to 10 show the separation of other breeds: BAR, CDD, TPR, MUR, NOR, MRF, and Napoletano (NAP). A radical majority of northern Italian coldblood breeds show earlier separation than warmblood breeds, which continue to be highly admixed until K = 19, where the warmblood horses of the Center and South Italy show a high percentage of admixture. Among them, Maremmano (MAR), Cavallo Romano della Maremma Laziale (CRM), Tolfetano (TOL) and Cavallo del Ventasso (CDV) are highly admixed and difficult to differentiate. In addition to MDS, structural analysis was repeated for the International dataset (Supplementary Figure S4), in this case for *K* values ranging from 2 to 39. For the International dataset, the CV value was the lowest at K = 39 (Supplementary Figure S5).

**FIGURE 4:**
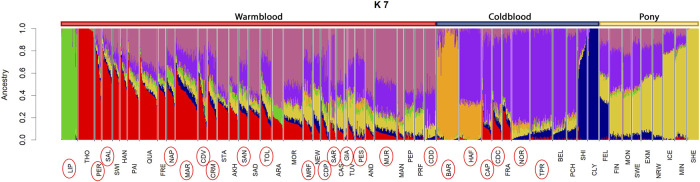
Admixture plot at K = 7 from the International dataset. On the top of the graph, color bars indicate groups of breed types: warmblood breeds are marked in red, coldblood breeds in blue and ponies in yellow. Full list of used acronyms is available in Supplementary Table S1.

At K = 2, we observed a separation between warmblood (in red, cluster referred to the Thoroughbred, THO) and coldblood (in blue, cluster referred to the Clydesdale horse, CLY) breeds. K = 3 discriminates the third group (in yellow), the pony cluster, referred to as the Shetland pony (SHE), mirroring what we also observed in the MDS. The LIP breed is again separated early at K = 4, and the first clustered Italian autochthonous breed, the BAR horse, emerges at K = 5. Belgian (BEL) and Arabian (ARA) horses were discriminated at K = 6 and K = 7, as shown in Figure 4. These two breeds, along with THO in the case of warmblood breeds, are among the most widespread genomic influences in the development of coldblood and warmblood breeds, respectively, and their lineages continue to be present at different percentages in all horse breeds; therefore, K = 7 was taken as an example for a complex but still clear overview of warmblood, coldblood, and pony group separation.

### Migration events

**FIGURE 5:**
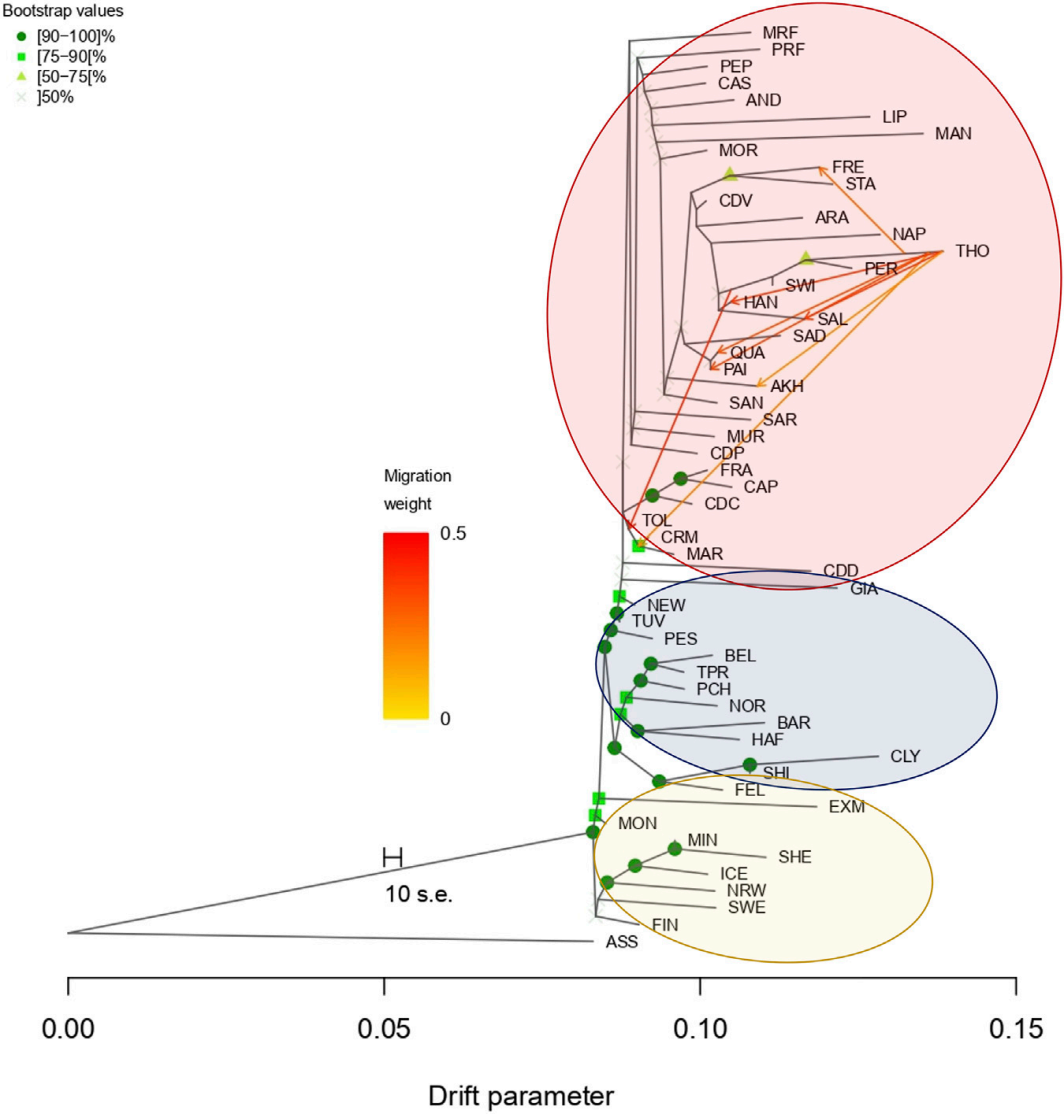
Gene flow analysis using TreeMix. Phylogenetic tree can only strongly validate coldblood breeds; gene flows are represented with arrows and the migration weight is differentiated by the color intensity. Warmblood, coldblood, and pony clusters are highly observable. Full list of used acronyms is available in Supplementary Table S1. Donkey population is identified with the acronym ASS.

The results obtained from the TreeMix software exploit the phylogenetic tree of all breeds. For coldblood and pony breeds (Figure 5) (lower part of the graph, blue and yellow, respectively), the software was able to strongly validate the phylogenetic relationships, while it was not possible (as expected) for the highly admixed warmblood populations (upper part, circled in red). The main result observed in this analysis is the wide introgression of Thoroughbreds in several warmblood breeds. As already stated, Thoroughbred have historically been used to improve other breeds for sport attitude and performance. Indeed, the gene flow has been found from Thoroughbred to Quarter Horse (QUA), Paint (PAI), French Trotter (FRE), Salernitano (SAL), Hanoverian (HAN), and MAR horses; a migration event from HAN to TOL horse is also present.

### Effective population size (N_e_)

**FIGURE 6:**
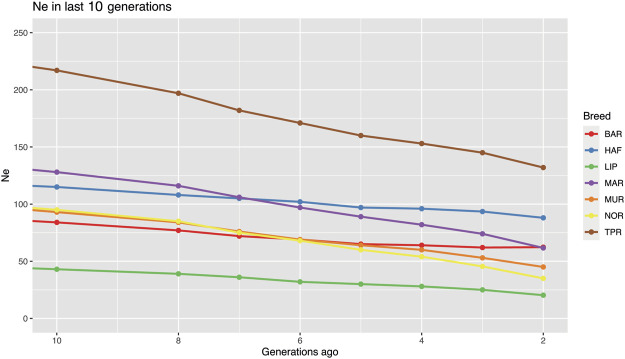
Effective population size estimation in seven Italian breeds (Bardigiano, BAR; Haflinger HAF; Lipizzaner, LIP; Maremmano, MAR; Murgese, MUR; Noriker, NOR; Cavallo Agricolo Italiano da Tiro Pesante Rapido, TPR) according to SNeP. Only last 10 generations are shown. Figure showing full analysis in the last 1,000 generations is available in Supplementary Figure S6.

Effective population size values were calculated only for the BAR, HAF, LIP, MAR, MUR, NOR, and TPR breeds, which satisfied the minimum consistency required. We can observe a very small Ne, especially for populations with low consistency, due to high inbreeding, bottlenecks, and/or geographical isolation. The LIP presented the lowest Ne due to the isolated breeding this breed experienced since its creation, also confirming previous analysis. Effective population analysis showed a low Ne in the seven Italian breeds considered. An inflection point dated roughly 450 generations ago was shown in all breeds as well as a rapid increase in the slope of the last 200 generations may be referred to the initiation of the domestication process (Supplementary Figure S6). Finally, a drastic reduction from about 40 to 13 generations ago was shown for most of the evaluated breeds (Supplementary Table S2). The current highest Ne resulted in the TPR breed both 10 and 2 generations ago (217 and 132, respectively). The lowest Ne was found in the LIP breed, which was 43 ten generations ago, and 20 in the most recent generation evaluated in this study. In the LIP breed, together with MAR and MUR, a drastic reduction was observed, with over 50% Ne reduction during the study period. In contrast, in the BAR and HAF breeds, although a reduction was observed, a more stable trend in Ne was noted (Figure 6).

## Discussion

Our results mirror what is historically reported and confirmed in previous studies on mitochondrial DNA of Italian horses (Cardinali et al., 2016). Italy has always been a hub across the Mediterranean Sea, both in North-to-South and in East-to-West directions. The genetic status of horses reflects this crossroad of cultures and population passage. We found a remarkably high level of admixture in the Italian breeds, mostly in the warmblood and southern populations, whereas the coldblood breeds in North Italy showed a more specific genetic identity. Indeed, Bardigiano, Haflinger, Noriker and Cavallo Agricolo Italiano da Tiro Pesante Rapido horses, alongside with Murgese, which shows more warmblood ancestry compared to the above-mentioned, are the Italian breeds that possess a distinct gene pool; the vast majority of warmblood breeds, on the other hand, has lower genetic identity and are mostly differentiated on their gradient of introgression with Thoroughbred/Arabian/Andaluso. Some breeds, such as Persano and Salernitano, Maremmano, Cavallo Romano della Maremma Laziale, Tolfetano, and Cavallo del Ventasso, were difficult to separate. Sanfratellano also does not appear to be a unique gene pool, as previously reported (Zuccaro et al., 2008). The only southern horse breed that appeared to have some degree of differentiation was Napoletano. However, this can be explained by the high founder effect and low breed consistency (census size <100 heads).

One of the reasons for the high admixture found in the majority of Italian warmblood is the strong introgression of thoroughbred lines applied to improve athletic and sport performances in the central and southern breeds. This was also demonstrated by TreeMix analysis, which confirmed genetic flows from the Thoroughbred to several breeds. On the Italian scale, the same dynamic can be hypothesized for the Maremmano breed (Giontella et al., 2019), which was also used in the development of Cavallo Romano della Maremma Laziale, Tolfetano, and Cavallo del Ventasso breeds, which clustered with Maremmano in all our analyses. Maremmano is indeed one of the major breeds in terms of consistency (approximately 8,000 individuals in the official Stud Book) and has a strong influence on the Italian warmblood gene pool (Giontella et al., 2020) with the exception of Salernitano and Persano breeds, which are virtually indistinguishable from each other and strongly influenced by Thoroughbreds. By definition, higher *K* values force the ADMIXTURE algorithm to find progressively new *K* groups, which makes it difficult (i.e., at K = 39) to appraise the three clusters (warmblood, coldblood, and ponies) at a glance. The peculiar differences in the population distribution (MDS) and genetic structure between warmblood and coldblood horses reflect the higher admixture and introgression of thoroughbred lines in warmblood breeds. In fact, Thoroughbreds have always been used to improve warmblood breeds and to enhance athletic qualities (Bailey et al., 2022); which is indeed visible in Italian breeds, where smaller populations needed this kind of improvement to get an economic and applicative relevance, especially in the last decades.

Different results can be found for the Cavallo del Delta and Lipizzaner breeds. These two breeds, particularly Lipizzaner, behaved as unique gene pools in national and international comparisons. The Cavallo del Delta is indeed a Camargue horse imported in Italy in the ‘70s, specifically in the river Po’s delta, which closely resembles the Rhone one (Bigi and Zanon, 2008). The Lipizzaner horse, on the other hand, has a very ancient and complex breed development history, which was established at the Habsburg court of Lipica (nowadays in Slovenia) in 1580 and since then was exposed to a closed studbook with few crossbred events (Kavar et al., 2002; Dovc et al., 2006). Lipizzaner is not strictly an Italian breed, but we included its peculiar gene pool within the Italian populations as one of the most important herds (Barcaccia et al., 2013) is preserved in Monterotondo (Rome) at CREA—*Consiglio per la ricerca in agricoltura e l’analisi dell’economia agraria* facility.

As already underlined, the Italian native breeds that show a clearer identity are coldblood Bardigiano and Haflinger horses. The Bardigiano breed, traditionally used in agriculture and meat production, is currently mainly used for riding and drafting. To help this transition, the Bardigiano Studbook routinely evaluates conformation and attitude traits based on a specific protocol with linear and traditional scoring systems to evaluate animals based on the breeding goal (Ablondi et al., 2020b). Thus, the use of this protocol over the last 30 years may have corroborated the distinctive gene pool found in this breed. Nevertheless, based on both pedigree and genotype data, alarming levels of inbreeding have been shown in the Bardigiano, highlighting the need for breeding strategies to ensure long-term survival (Ablondi et al., 2018; Ablondi et al., 2020a). Therefore, based on the peculiar gene pool shown in this study and the previous characterization of within-breed diversity, we recommend the further implementation of breeding strategies to preserve this local heritage. Another well-recognized coldblood breed is the Italian Heavy Draught Horse or Cavallo Agricolo Italiano da Tiro Pesante Rapido. The breed originated in half of the 19th century, when the Kingdom of Italy was established, with the purpose of getting a heavy horse for rapid draught to be used in agricultural work and field artillery (Mancin et al., 2020). After some first attempts to cross coldblood mares native to the northeast of Italy with stallions from the most famous coldblood breeds, such as Percheron and Belgian (traces of these crossbreeding practices can be seen in the structure analysis), the breed originated by using French stallions of Norfolk-Breton. The population has experienced a strong decline in head counts since the 1960s due to the expansion of mechanization in agriculture, but it has survived thanks to the introduction of meat production as additional breeding purpose. Nowadays, the original heavy-draught attitude has reached a new interest in the activities of holiday farms, tourism, and leisure (Mancin et al., 2020). Another coldblood breed is the Noriker, native to Celtic settlements in the Alpine valleys in Austria and Italy. The genetic improvement of the breed started in the 16th century, when selected coldblood stallions were used, including Haflinger, Napoletano, and Andaluso (Druml et al., 2008). Over time, the “heaviest” animals were selected for use in draught and riding at work and in sports activity; even after the Second World War, a small but deep-chested animal was preferred. The breed experienced a decline in the second half of the 20th century, similar to Cavallo Italiano da Tiro Pesante Rapido, but it did not disappear because of the characteristics of mild temperament and power that are distinctive of the breed.

Another breed that represents a unique gene pool in the Italian equine landscape is the Murgese. The breed shows an early separation in the ADMIXTURE analysis (K = 7 and K = 11 in the Italian and International dataset, respectively), and a distinct behavior in the MDS examining D3 and D4 (Italian dataset) or in D2 *vs*. D6 in the International, in which the breed is located in the “Arabian cluster” as previously observed with uniparental data (Cardinali et al., 2016).

The effective population size analysis showed a low Ne in the seven Italian breeds considered. The inflection point shown at 450 generations ago may be the result of the initiation of the domestication process. Whereas the rapid increase in the slope of the last 200 generations might represent a bottleneck due to the first selection process in ancient times when horses became an active part of human activities. Finally, a drastic reduction from about 40 to 13 generations ago can represent the bottleneck this species went into during the industrial era after the progressive reduction of its working role, the advent of genetic improvement activities, and the use of a few top-ranked stallions for reproduction. In the last ten generations, all major Italian breeds showed a reduction in Ne. The highest Ne resulted in the TPR breed both ten and two generations ago (217 and 132, respectively).

In conclusion, the progressive decrease in population size in the last decades, due to the cessation of their traditional role in society and the suffering and struggling of the equine sport industry, led to an important reduction in equine genetic diversity, confirmed by effective population size analysis. Hence, it is fundamental to provide novel strategies to be able to preserve the national breeds in the long term. Some efforts in this direction are already underway in some breeds as for example the Bardigiano breed, with early modern monitoring of inbreeding and biodiversity reduction. Finally, we can state that the recognition of some of the Italian horse populations as breeds mirrors cultural and regional traditions rather an actual genetic differentiation. Extensive knowledge of Italian equine genetic makeup represents an essential starting point for developing conservation strategies and breed management. The results of this study can be used to guide stakeholders and politicians to better employ resources for conservation programs and appraise, at different levels (scientific, cultural, and historical), the Italian equine genetic heritage.

## Data Availability

Data are available upon reasonable request to interested researchers from the corresponding authors.
