# Supplementary material for: Exploring the Italian equine gene pool via high-throughput genotyping
Source: Front Genet. 2023 Jan 23;14:1099896. doi: 10.3389/fgene.2023.1099896 (PMC9900106; doi:10.3389/fgene.2023.1099896)
Supplement: Supplementary file 1 [file DataSheet1.pdf]

Supplementary Material

1.1 Supplementary Figures

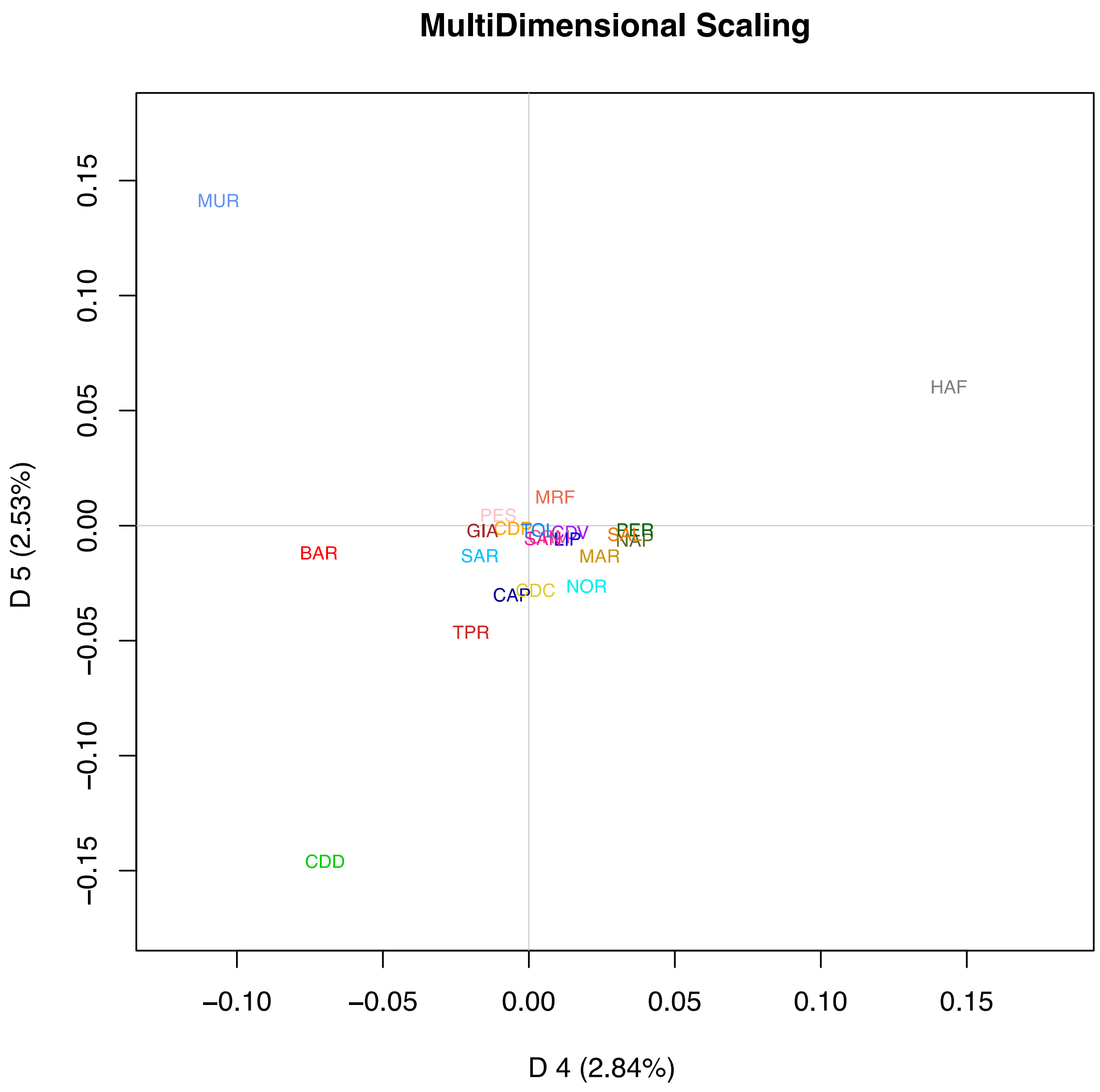

Supplementary Figure 1. Dimension 4 and Dimension 5 MDS plot from the Italian dataset analysis where further separation of MUR (Murgese) and CDD (Cavallo del Delta) can be appraised. Average

coordinates for each breed are plotted. Full list of used acronyms is available in Supplementary Table 1.

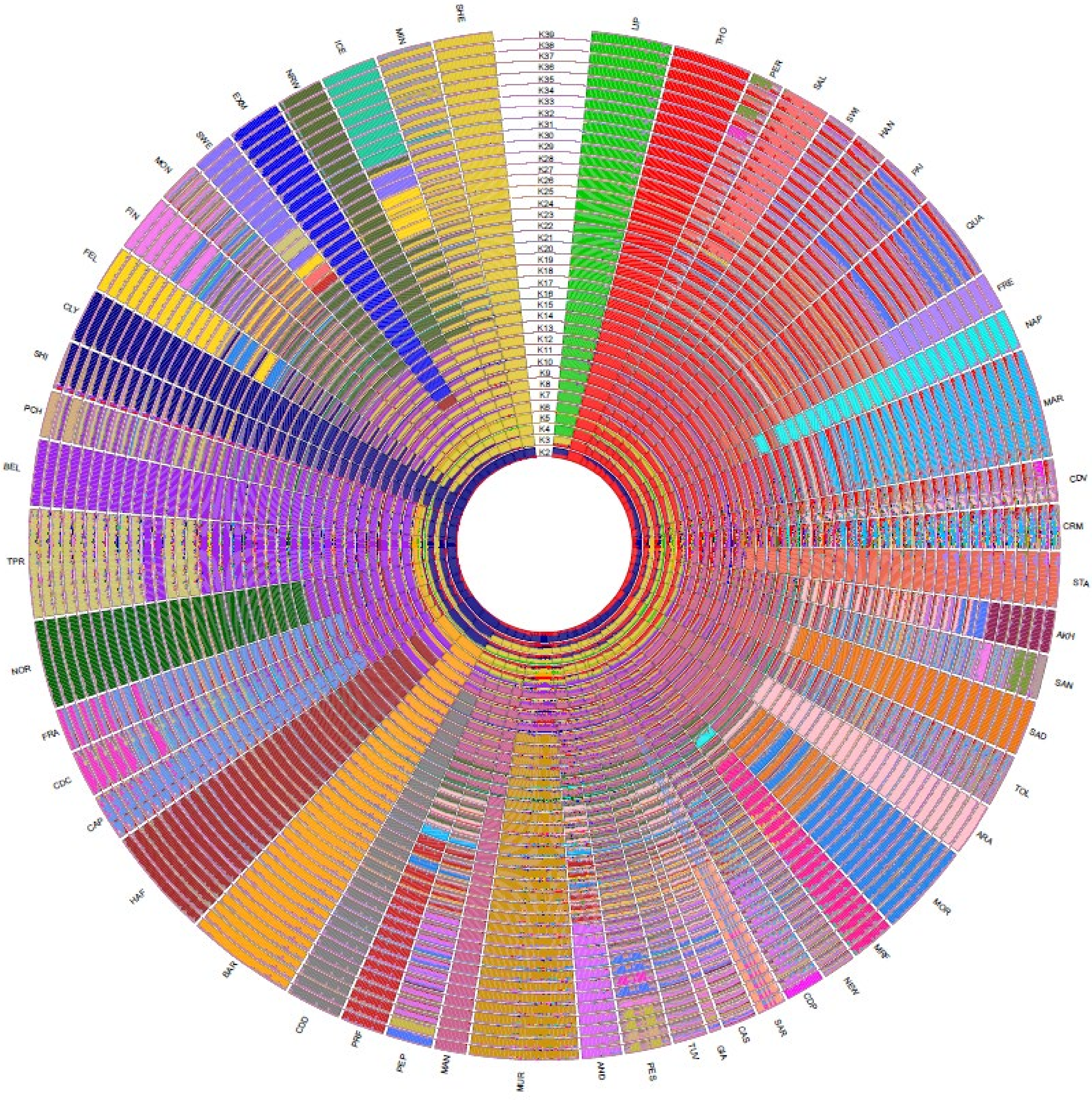

Supplementary Figure 2. Structure analysis for the international dataset from K2 to K39. Full list of used acronyms is available in Supplementary Table 1.

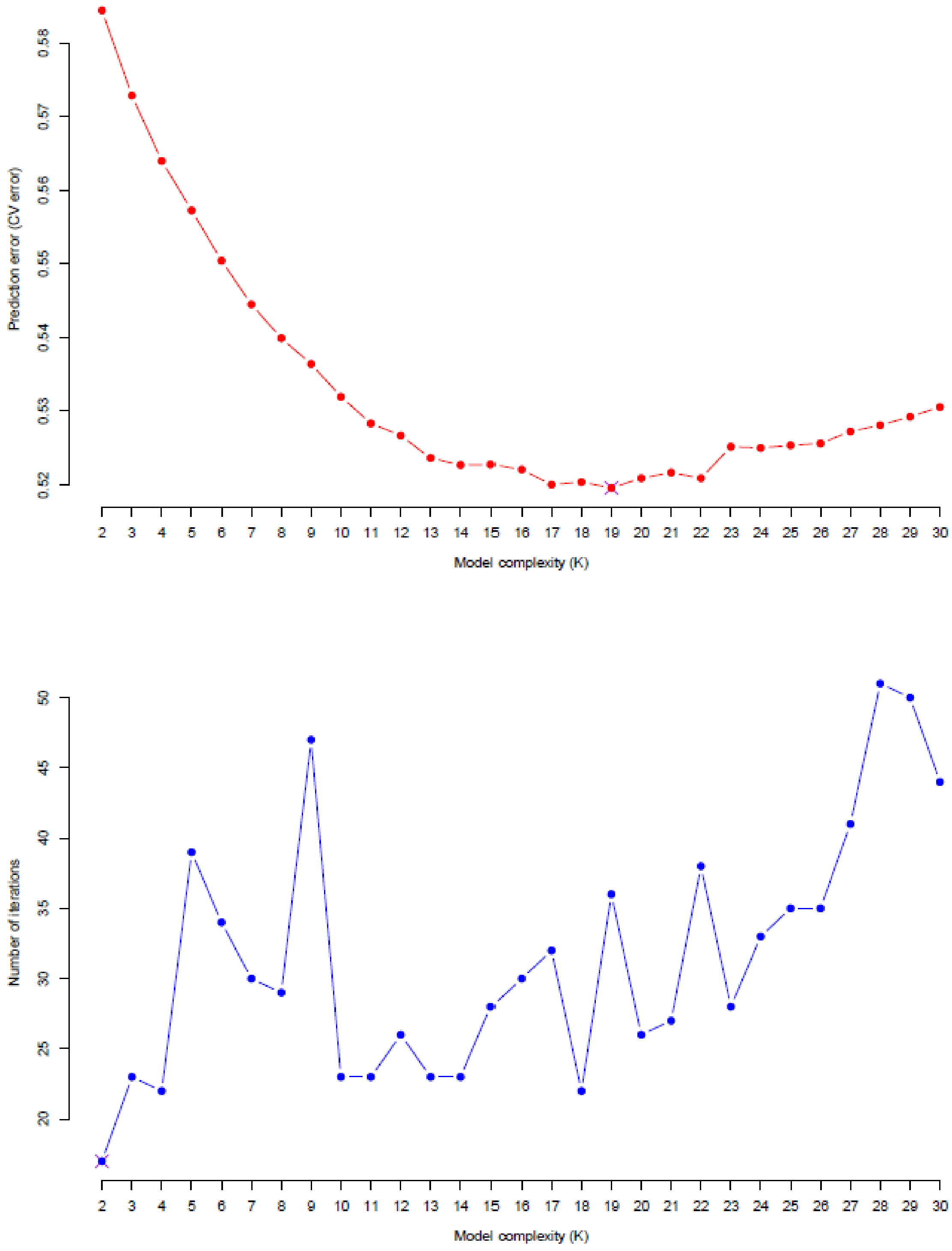

Supplementary Figure 3. Cross-validation procedure in the Italian dataset to estimate the accuracy of ancestry percentages: the lowest estimated value of cross-validation (CV) error is associated with the most fitting value of ancestral genomes.

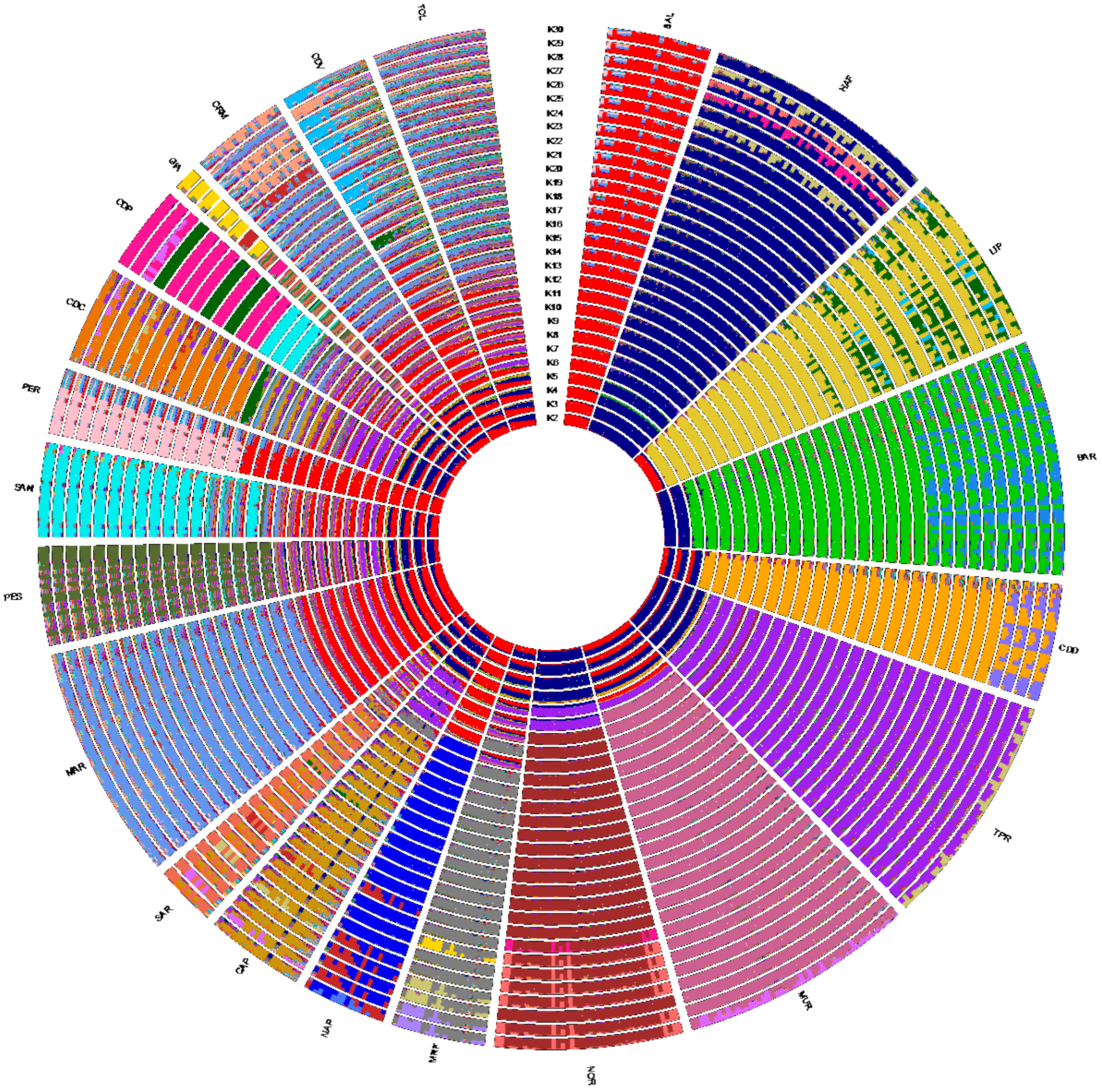

Supplementary Figure 4. Structure analysis for the Italian dataset from K2 to K30. Breeds are ordered contemplating clustering solutions. Full list of used acronyms is available in Supplementary Table 1.

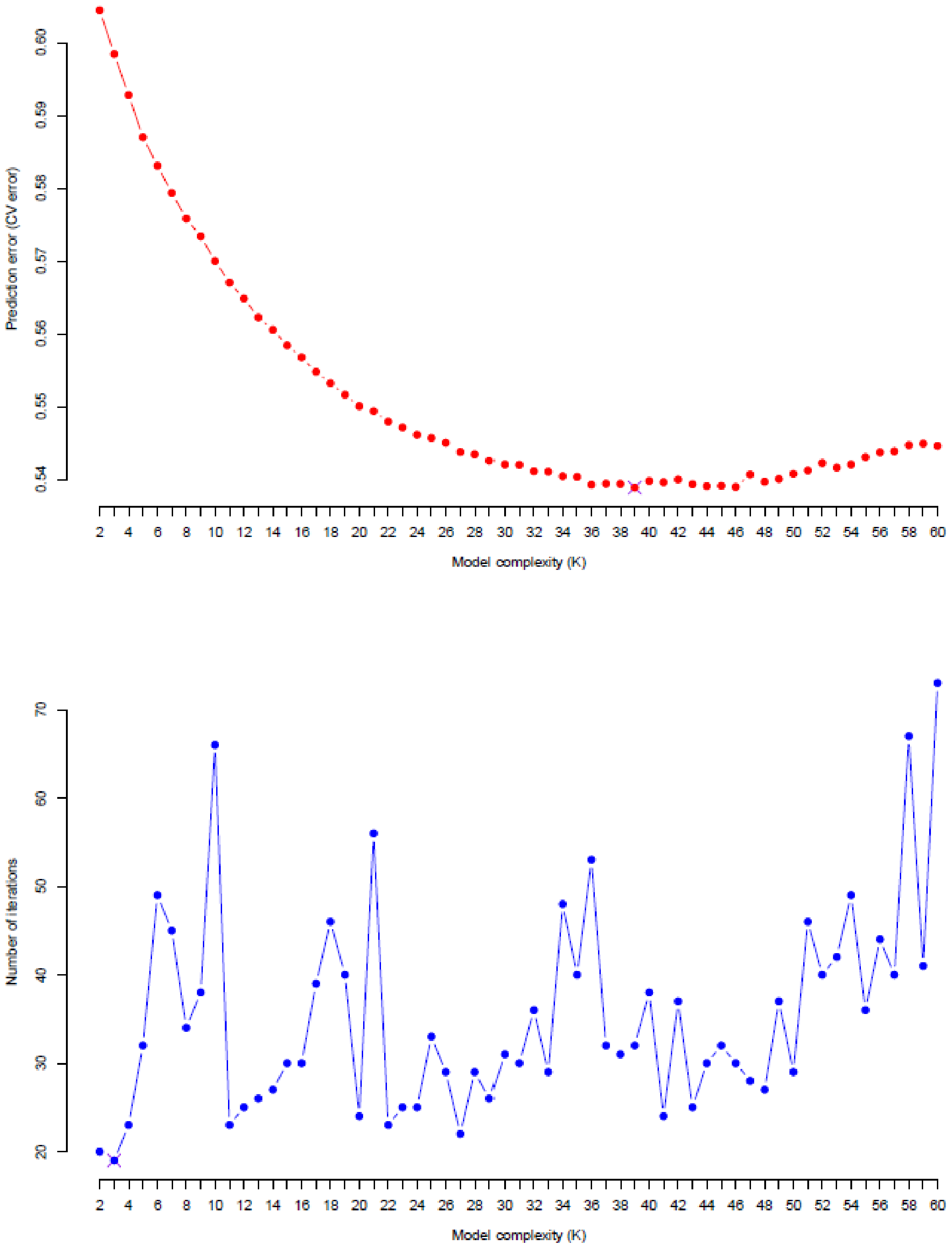

Supplementary Figure 5. Cross-validation procedure in the cosmopolitan dataset to estimate the accuracy of ancestry percentages: the lowest estimated value of cross-validation (CV) error is associated with the most fitting value of ancestral genomes

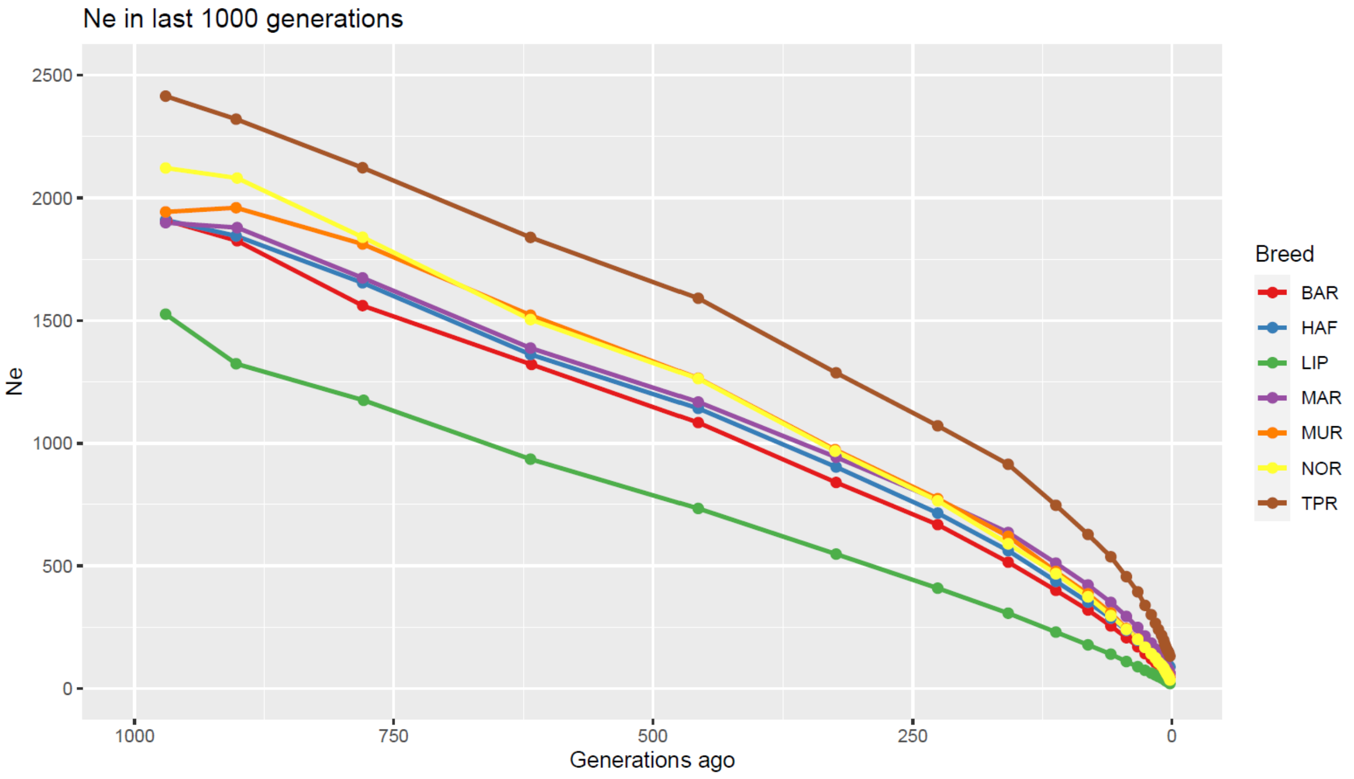

Supplementary Figure 6. Effective population size estimation in seven Italian breeds (Bardigiano, BAR; Haflinger HAF; Lipizzaner, LIP; Maremmano, MAR; Murgese, MUR; Noriker, NOR; Cavallo Agricolo Italiano da Tiro Pesante Rapido, TPR) according to SNeP in the last 1000 generations.

1.2 Supplementary Tables

Supplementary Table 1. Samples used in the study with consistencies pre and post quality control.

BREED	ACRONYM	SAMPLING REGION	DATA ORIGIN	N° OF INDIVIDUALS	CONSISTENCY AFTER QC AND SUBSAMPLING
**AKHAL TEKE**	AKH	US & Russia	Petersen *et al.*, 2013	20	19
**ANDALUSIAN**	AND	United States	Petersen *et al.*, 2013	18	18
**ARABIAN**	ARA	United States	Petersen *et al.*, 2013	24	24
**BARDIGIANO**	BAR	ITALY	This study	185	50
**BELGIAN**	BEL	United States	Petersen *et al.*, 2013	30	30
**CAVALLO APPENNINICO**	CAP	ITALY	This study	21	21
**CASPIAN**	CAS	United States	Petersen *et al.*, 2013	16	15
**CAVALLO DEL CATRIA**	CDC	ITALY	This study	23	21
**CAVALLO DEL DELTA**	CDD	ITALY	This study	25	25
**CAVALLO PENTRO**	CDP	ITALY	This study	18	18
**CAVALLO DEL VENTASSO**	CDV	ITALY	This study	19	19
**CLYDESDALE**	CLY	US & UK	Petersen *et al.*, 2013	24	24
**CAVALLO ROMANO DELLA M. LAZIALE**	CRM	ITALY	This study	20	20
**EXMOOR**	EXM	United Kingdom	Petersen *et al.*, 2013	24	24
**FELL PONY**	FEL	United Kingdom	Petersen *et al.*, 2013	21	21
**FINNHORSE**	FIN	Finland	Petersen *et al.*, 2013	27	27
**FRANCHES MONTAGNES**	FRA	Switzerland	Petersen *et al.*, 2013	19	19
**FRENCH TROTTER**	FRE	France	Petersen *et al.*, 2013	17	17
**CAVALLINO DELLA GIARA**	GIA	ITALY	This study	5	5
**HAFLINGER**	HAF	ITALY	This study	235	50
**HANOVERIAN**	HAN	Germany	Petersen *et al.*, 2013	15	15
**ICELANDIC**	ICE	Sweden	Petersen *et al.*, 2013	25	25
**LIPIZZAN**	LIP	ITALY	This study	40	37
**MANGALARGA PAULISTA**	MAN	Brazil	Petersen *et al.*, 2013	14	14
**MAREMMANO**	MAR	ITALY	This study	90	50
**MINIATURE**	MIN	United States	Petersen *et al.*, 2013	25	25
**MONGOLIAN**	MON	Mongolia	Petersen *et al.*, 2013	19	19
**MORGAN**	MOR	United States	Petersen *et al.*, 2013	43	43
**MONTERUFOLINO**	MRF	ITALY	This study	25	20
**MURGESE**	MUR	ITALY	This study	60	50
**NAPOLETANO**	NAP	ITALY	This study	18	18

**NEW FOREST PONY**	NEW	United Kingdom	Petersen *et al.*, 2013	15	15
**NORIKER**	NOR	ITALY	This study	40	40
**NORWEGIAN FJORD**	NRW	Norway	Petersen *et al.*, 2013	21	21
**PAINT**	PAI	United States	Petersen *et al.*, 2013	25	25
**PERCHERON**	PCH	United States	Petersen *et al.*, 2013	22	21
**PERUVIAN PASO**	PEP	United States	Petersen *et al.*, 2013	21	21
**PERSANO**	PER	ITALY	This study	14	14
**PONY ESPERIA**	PES	ITALY	This study	21	21
**PUERTO RICO PASO FINO**	PRF	Puerto Rico	Petersen *et al.*, 2013	20	20
**QUARTER HORSE**	QUA	United States	Petersen *et al.*, 2013	40	40
**SADDLEBRED**	SAD	United States	Petersen *et al.*, 2013	25	25
**SALERNITANO**	SAL	ITALY	This study	26	22
**SANFRATELLANO**	SAN	ITALY	This study	22	20
**SARCIDANO**	SAR	ITALY	This study	13	13
**SHETLAND**	SHE	Sweden	Petersen *et al.*, 2013	27	27
**SHIRE**	SHI	United States	Petersen *et al.*, 2013	23	22
**STANDARDBRED**	STA	US & Norway	Petersen *et al.*, 2013	25	25
**NORTH SWEDISH HORSE**	SWE	Sweden	Petersen *et al.*, 2013	19	19
**SWISS WARMBLOOD**	SWI	Switzerland	Petersen *et al.*, 2013	14	14
**THOROUGHBRED**	THO	US, UK & Ireland	Petersen *et al.*, 2013	36	36
**TOLFETANO**	TOL	ITALY	This study	25	24
**CAVALLO AGRICOLO ITALIANO DA TIRO PESANTE RAPIDO**	TPR	ITALY	This study	363	50
**TUVA**	TUV	Russia	Petersen *et al.*, 2013	15	15
**TOTAL**				**2037**	**1333**

Supplementary Table 2. Inbreeding (F) based on loss of heterozygosity and Effective population size (Ne) for breeds with at least 40 animals genotyped.

	F calculated on subset database (N=608)					F calculated on whole database after QC (N=1269)					Ne		
BREED	*N.*	MEAN	MIN	MAX	SD	*N.*	MEAN	MIN	MAX	SD	GEN 20	GEN 10	GEN. 2
BAR	*50*	0.17	0.08	0.35	0.05	*183*	0.14	-0.06	0.34	0.05	121	84	62
CAP	*21*	0.11	-0.07	0.23	0.08	*21*	0.08	-0.10	0.21	0.08			
CDC	*21*	0.05	-0.07	0.13	0.05	*21*	0.02	-0.11	0.10	0.05			
CDD	*25*	0.17	0.08	0.29	0.06	*25*	0.15	0.05	0.27	0.06			
CDP	*18*	0.09	0.06	0.14	0.03	*18*	0.06	0.03	0.12	0.03			
CDV	*19*	-0.02	-0.07	0.18	0.06	*19*	-0.05	-0.10	0.15	0.06			
CRM	*20*	0.00	-0.06	0.11	0.05	*20*	-0.03	-0.09	0.08	0.05			
GIA	*5*	0.14	0.10	0.25	0.06	*5*	0.12	0.07	0.22	0.07			
HAF	*50*	0.15	0.09	0.24	0.04	*219*	0.14	0.06	0.25	0.04	148	115	88
LIP	*37*	0.16	0.09	0.23	0.04	*37*	0.13	0.06	0.20	0.04	63	43	20
MAR	*50*	-0.01	-0.07	0.12	0.03	*86*	-0.04	-0.11	0.09	0.04	185	128	62
MRF	*20*	0.01	-0.04	0.24	0.06	*20*	-0.02	-0.07	0.22	0.06			
MUR	*50*	0.07	0.02	0.15	0.03	*60*	0.05	-0.01	0.13	0.03	141	93	45
NAP	*18*	0.03	-0.06	0.21	0.07	*18*	0.01	-0.09	0.19	0.07			
NOR	*40*	0.15	0.06	0.21	0.03	*40*	0.12	0.03	0.19	0.03	145	105	62
PER	*14*	0.01	-0.07	0.30	0.10	*14*	-0.02	-0.10	0.28	0.10			
PES	*21*	0.10	-0.01	0.22	0.05	*21*	0.08	-0.05	0.19	0.05			
SAL	*22*	-0.02	-0.12	0.03	0.04	*22*	-0.05	-0.15	0.00	0.04			

SAN	*20*	0.04	-0.01	0.12	0.04	*20*	0.01	-0.04	0.09	0.04			
SAR	*13*	0.16	0.02	0.26	0.06	*13*	0.14	-0.01	0.24	0.07			
TOL	*24*	0.00	-0.08	0.18	0.05	*24*	-0.03	-0.11	0.16	0.06			
TPR	*50*	0.11	0.07	0.17	0.02	*362*	0.09	0.02	0.37	0.04	301	217	132
